# The coverage of environmental issues in FM radios in Nepal: the current status and challenges

**DOI:** 10.1016/j.heliyon.2020.e04354

**Published:** 2020-07-03

**Authors:** Prakash Kumar Paudel, Rabin Bastola, Pashe Tamang Lopchan

**Affiliations:** a Center for Conservation Biology, Kathmandu Institute of Applied Sciences, Kathmandu, Nepal; b Department of Environmental Science, Amrit Campus, Tribhuvan University, Kathmandu, Nepal; c Sampanna Sanchar Pvt. Ltd., Kathmandu, Nepal; d Ujyaalo Radio Network, Lalitpur, Nepal

**Keywords:** Social science, FM radio, Mass media, Environmental communication, Community awareness, Online survey, Media strategy

## Abstract

Radio stations particularly those of local FM have an influential role in disseminating environmental concerns among the wide populace. This study provides the first nationwide assessment of FM radio coverage of environmental issues in Nepal by using data collected from a web-based questionnaire survey from 102 radio stations. We found that environmental programs were covered in 57% of radio stations, but they were among the least popular ones. Among various categories of environmental programs, the general category was the most common and popular, followed by forest and wildlife conservation and water resource management. Surprisingly, environmental pollution and disaster risk management were ranked as being less popular in comparison to their coverage. Environmental programs were mostly broadcasted weekly (73%) during prime time (morning and evening). Although there were agreements among respondents on the importance of environment program for radio stations (agreement = 0.74), nearly 22% of all radio stations terminated such programs in the past and only a few of them were interested to relaunch them in the foreseeable future. Limited funding was the most reported challenge followed by difficulty in finding environmental experts and radio journalists. We recommend the Government of Nepal to formulate a media engagement strategy to strengthen environmental campaigns in the face of rapid environmental change in Nepal.

## 1 Introduction

Mass media have been a critical avenue to reach, communicate, and influence people on the alarming intensity and frequency of environmental problems [1, 2, 3]. The world is facing a multitude of environmental problems, ranging from biodiversity loss, environmental pollution, spread of the vector-borne diseases and climate change at varying spatial and temporal scales [4]. Many environmental problems are results of human activities—both intentional and unintentional — that eventually either drive or trigger environmental catastrophes [5]. A better understanding of such problems and their consequences is crucial to change public opinion and garner public support for environmental protection [3, 6, 7].

Environmental issues started becoming national and global concerns since the 1960s, thanks to media [8], with the publication of Rachel Carson's *Silent Spring* [9], Chernobyl nuclear disaster [10] and Bhopal disaster [11]. Now, environmental communication has become widespread and complex, so has been people's participation in environmental movements throughout the world [12]. These changes are brought by advent of the media, including an expansion of international organizations and increased global connectivity (e.g., airways, waterways, etc.) [13, 14]. There is a need for concerted and coordinated efforts at both global and local levels for developing tailored made multilevel tasks [4, 12]. Here, local mass media play critical roles in both informing people about global and local environmental issues and stimulating public engagements [3, 15]. However, research suggests that the effectiveness of environmental communication depends on access to media, frequency and coverage of issues, level of interactions and engagements, trained journalists and availability of experts [15, 16, 17, 18].

Environmental communication is a crisis discipline that educates, alerts, persuades and helps to solve environmental problems where information follows through different communication channels [18]. Thus, developing messages, selecting appropriate media and reaching an audience are important aspects of the communication strategy [16]. Mass media—– television, radio, newspapers, pamphlets, fact sheets, billboards, magazines, the Internet, etc.—are means of informal education [19]. Radios, in particular, are popular in rural areas as they are cheap, convenient to use in various workstations, make connections with people through local language, music and news [20, 21, 22, 23]. They are primary sources of information in the developing countries [25, 26]. This is true for Nepal where 80% people still use radios as a primary source of information [27]. Nepal made a great stride in promoting radio channels, mostly FM radios that successfully established as a mainstream media with restoration of liberal democracy in 1990 and contributed to the promotion of local languages and cultures, dissemination of news in remote areas [28]. Currently, a total of 740 licenses are issued to FM radio stations (available at http://mocit.gov.np/detail/cable-tv-and-fm). The licensees include nongovernment organizations (NGOs), cooperatives and local government bodies, known as *community radio*, and commercial entities, known as *commercial radio* [29]. The community FM radios became voices of communities on their social, political, economic and cultural concerns, whereas commercial FM radios created a unique forte through listeners' participation [30].

Nepal is one of the most vulnerable regions of the world to global environmental change and disasters [31, 32]. A rapid environmental change coupled with ill-planned developments has already put the country at risk of disasters [33, 34]. Here, FM radios will remain a mainstream media and play important roles in environmental communication given their high coverage in the rural areas and availability of inexpensive radio sets. A better understanding of radio coverage of environmental issues and challenges therein is important to develop a media strategy [3, 17, 35, 36].

The inclusion of environmental programs in media and their effectiveness depends on various factors such as funding, human resources and people's interest. Radio stations sustain largely from advertisements and airtime sponsors from business enterprises [37]. Funding for the environmental program comes mostly from organizations—government and non-government— for public interest, whereas some radio stations prioritize environmental programs as part of their corporate social responsibilities. Business enterprises focus on popular programs such as entertainment (e.g., songs, music) and political programs which are often well-received by the public. Besides funding, trained environmental journalists are critical to run environmental programs [38].

Our research thus has the following six research questions:

1. To what extent (e.g., frequency, time and duration) environmental programs are included in the FM radios, and whether community and commercial radio stations differed significantly?
2. Which categories of environmental programs (general, water resource management, disaster risk management, climate change, forest and wildlife conservation and environmental pollution) are the most common? Are common programs popular?
3. Are audience feedback (e.g., phone, survey, email) and popularization (e.g., radio jingle) mechanism used in environmental programs?
4. What are the perceived impacts of environmental programs at the local level?
5. What are the major challenges of running environmental programs and why some radio stations terminated their environmental programs in the past?
6. From where funding for the environmental program comes?

Our study, as such breadth and coverage, is the first one in the region. Such information is important to develop a media strategy in Nepal.

## 2 Materials and methods

### 2.1 Ethical statement

This research is supported by the Kathmandu Institute of Applied Sciences (KIAS). We submitted survey questionnaires to the Research Ethics Committee (REC) at Kathmandu Institute of Applied Sciences (KIAS) for ethical approval. REC determined that the survey do not have ethical issues and that confidentiality of respondents and their responses were determined, and informed consent was sought (ref number: RE-18-01).

### 2.2 Survey design and sampling

We surveyed FM radios using a proprietary software SoGoSurvey (SoGo Survey Inc., Herndon, VA; www.sogosurvey.com). There is a total of 740 licenses issued to radio channels. Some of them ceased broadcasting while others did not have valid contact information such as phone and email. We finally collected (a) name, (b) address, and (c) email address of 398 radio stations from the Ministry of Information and Communication (now Ministry of Communication and Information Technology), Association of Community Radio Broadcasters Nepal (ACORAB) and Ujyaalo 90 Network. We emailed invitations between 30 April 2018 to 21 July 2018 using SoGoSurvey platform with a secure link for questionnaires, which enabled us to check progress and make a strategy for reminder emails. We explicitly sought participation of station managers who have broader understanding of programs conducted in their respective radio stations. We sent a maximum of three reminder emails, and follow up calls were made to non-respondents. In total, 102 emails were responded, resulting in a response rate of 25%.

### 2.3 Questionnaire

The questionnaire included tailored made questions depending on responses of previous questions using conditional branching. The survey included Likert scale, multiple-choice, ranked and open questions. The first part of questionnaire included general questions about radio frequency, coverage, audience number, broadcasting time and association with radio network. The second part of questionnaire covered information about most popular programs (excluding news), including environmental programs. We used various categories of regular programs: economy, entertainment, environment, health, politics, science and technology, society, sports. Environmental programs were further investigated under following categories: environmental pollution, forest and wildlife conservation, climate change, disaster risk management, water resource management and general. Those radio stations that discontinued environmental programs were asked to choose major reasons, with an option of adding any others. Radio channels that air environmental programs were asked about human resources, type of environmental programs and their broadcasting time, frequency and duration. Several additional questions were asked at the end (see Appendix 1).

### 2.4 Data analysis

We computed descriptive statistics (mean ± SD, median) of network, audience number, broadcast time of radio stations by various categories. We calculated frequencies of top five radio programs, including those radio stations airing environmental programs. We used Pearson's chi-square test to analyze the difference in frequencies among different categories of (a) radio programs (economy, entertainment, environment, health, politics, science and technology, society, sports and all) and (b) environmental programs (e.g., water resource management, forest and wildlife conservation, environmental pollution, climate change, disaster risk reduction). The Mann–Whitney U-test was used to compare audience number and broadcast time between public and commercial radio stations. Kruskal-Wallis H test was used to compare ranks of different funding sources. Post-hoc multiple comparison tests (Dunn's tests) were subsequently used separately to determine funding sources that differ from others. Proportions were compared using the two-proportion z-test. We computed popularity indices of environmental programs to check their conformity with respondents' ranks as:

Popularityindex=(primetime+frequencyscore+duration), where prime time (6:00–8:00; 18:00–20:00) and non-prime time were 4 and 2 respectively. Frequency score of each of environmental program assigned as 30/37 for daily, 4/37 for weekly, 2/37 for fortnightly and 1/37 for monthly, where 37 is maximum frequencies of environmental programs in a month (e.g., 30+4+2+1). Duration is number of years of continuous broadcasting of environmental programs.

All analyses were carried out in R v1.2.1335 [39] using package ‘pastecs’ [40] for descriptive statistics, ‘ggplot2’ [41], ‘likert’ [42], and ‘ggsignif’ [43] for analyses and graphical presentation of bar diagram, including Tableau (version 2019.1). We used function ‘kruskal.test’ of R base and ‘DunnTest’ of package ‘FSA’ [44] to carry out non-parametric Kruskal Wallis test and its post-hoc comparisons. Thus, data analyses, interpretations and conclusions are limited by the type of respondents participated in the survey and their responses.

## 3 Results

A total of 102 radio stations participated in the survey, which included community radio stations (59%) and commercial radio stations (41%). These radio stations represented a large variability in terms of coverage, audience size, broadcast time and years in operation (Table 1). Most of the radio stations were associated with networks (93%), which included the Association of Community Radio Broadcasters Nepal (ACORAB) (38%), Ujyaalo 90 Network (30%), Other (25%) and Nepal FM (7%). The radio stations covered 1 to 75 districts (100% of Nepal) with a mean of 9.05 ± 9.42 and a median of 7 districts through its own network, and 13.06 ± 20.25 and a median of 6 districts from associated radio network. An estimated audience of each radio station ranged from less than one hundred thousand to five million (Table 1) with an average 557 thousand. Radio stations were established from 1998 to 2017, with median year of 2011. Almost all the radio stations had internet live stream (92%), which included commercial radio (95%), community radio (92%). Only 57% radio stations covered environmental issues, which included community (65%) and commercial radio stations (57%).

**Table 1:** General characteristics of community, commercial and all radio stations participated in the survey.

Category	Mean	Median	SD
All radio station (n = 102)			

Number of districts covered through own network	9.05	7	9.42
Number of districts covered through associated network	13.06	6	20.25
Number of audience (hundred thousand)	5.57	3.25	6.82
Radio broadcast time (hours)	17.99	18	1.57

Community radio stations (n = 65)			

Number of districts covered through own network	9.21	7	10.94
Number of districts covered through associated network	12.20	6	19.64
Number of audience (hundred thousand)	5.58	3	7.23
Radio broadcast time (hours)	17.58	18	10.59

Commercial radio stations (n = 37)			

Number of districts covered through own network	8.78	8	6
Number of districts covered through associated network	14.59	6	21.48
Number of audience (hundred thousand)	5.55	3.75	6.04
Radio broadcast time (hours)	18.70	18	2.02

Radios that broadcast environmental program (n = 58)			

Number of districts covered through own network	9.27	7	11.01
Number of districts covered through associated network	14.15	6.5	20.59
Number of audience (hundred thousand)	3.5	6.1	7.77
Radio broadcast time (hours)	17.89	18	1.60

Radios that do not broadcast environmental program (n = 40)			

Number of districts covered through own network	7.5	8.77	6.88
Number of districts covered through associated network	11.63	5	19.94
Number of audience (hundred thousand)	4.83	2.75	5.40
Radio broadcast time (hours)	18.11	18.00	1.52

Of 102 respondents, the majority were executives and/or senior managers (77%) and officers (23%). Almost all of respondents were male (96%). Work experience of respondents ranged from 1 to 20 years, with a mean 8 years. Nearly 33% of respondents had master's degrees, and 10% had bachelor's degrees while the rest of them had education status below the undergraduate level.

### 3.1 Coverage and popularity of radio programs

#### 3.1.1 Overall radio programs

**Figure 1:**
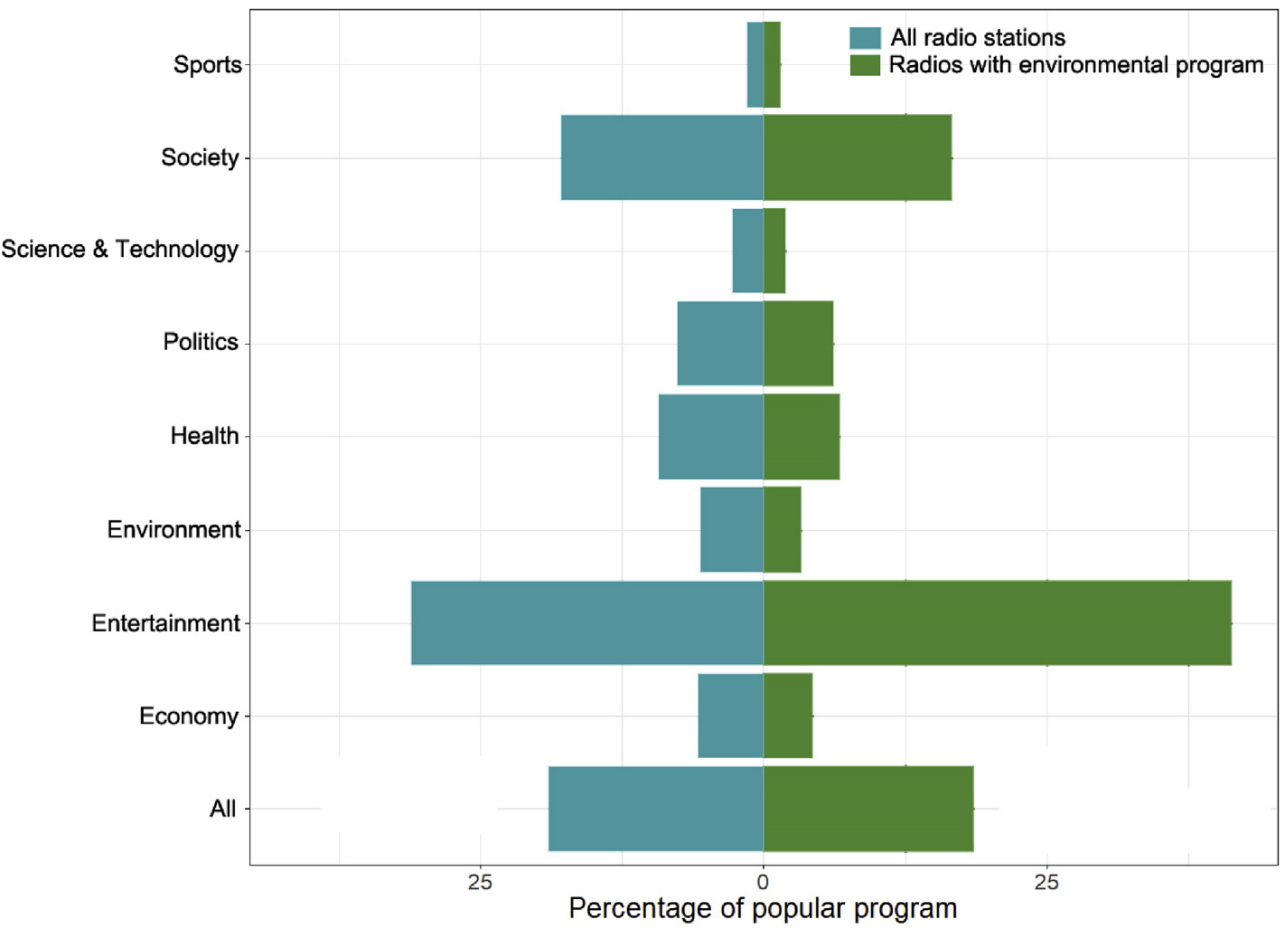
Percentage of the top five most popular programs aired by all radio stations (left) and radio stations that covers environment related programs (right).

The top five most popular radio programs were on entertainment, followed by mixed ones, society, health, politics, economy and environment. Science and technology and sports were among the least popular programs (Figure 1). Overall, there were significant differences among various categories of programs aired by radios (X^2^ = 325.38, df = 8, P < 0.001). The radio stations that covered environment related programs also showed the same pattern (X^2^ = 251.16, df = 8, P < 0.001). As expected, a significantly different pattern was observed between community and commercial radio stations (X^2^ = 42.61, df = 8, P < 0.001). Community radio stations covered more programs on society (23%) and environmental issues (4%) in comparison with commercial radio stations, which had 17% programs on society and just 0.8% programs on environmental issues. Overall, environment related programs were among the least common in both types of radio stations, just better than science & technology and sports (Figure 1).

#### 3.1.2 Environmental programs

**Figure 2:**
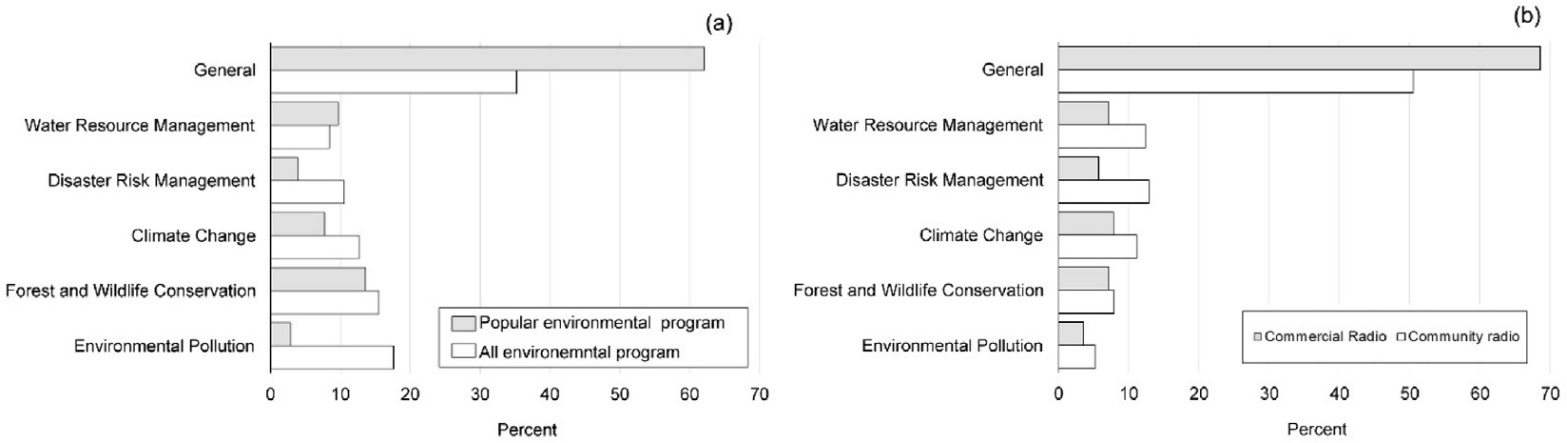
(a) Percentage of coverage of various categories of environmental programs and their popularity among 102 radio stations in Nepal (b) Percentage of coverage of environmental programs by community and commercial radio stations.

Environmental programs were aired by 57% of radio stations. These radio stations covered average 9.27 ± 11.01 districts through own network and 14.15 ± 20.59 districts through associated networks. Only 43% of commercial radio stations and 65% of community radio stations aired environmental programs (Table 1). There were significant differences in total numbers among different categories of environmental programs for all radio stations (X^2^ = 467.52, df = 4, P < 0.001), commercial radios (X^2^ = 220.59, df = 4, P < 0.0001) and community radios (X^2^ = 268.03, df = 4, P < 0.0001). The most common environment related programs were on general issues (35%), followed by environmental pollution (18%), forest and wildlife conservation (15%), climate change (13%) and disaster risk management (11%) (Figure 2) (see Appendix 2 for a list of some environmental programs). But popular programs differed significantly from total environmental programs (X^2^ = 25.62, df = 5, P < 0.0001). Environmental programs covering all issues (general category) were both the most common and popular. Environmental programs covering environmental pollution and disaster risk management were ranked as being less popular in comparison to their coverage (Figure 2). The popularity index of various categories of environmental programs was correlated significantly with their respective ranks by respondents (F = 1980, R = 0.99, P < 0.0001).

### 3.2 The pattern of environmental program: duration, frequency, and environmental events

**Figure 3:**
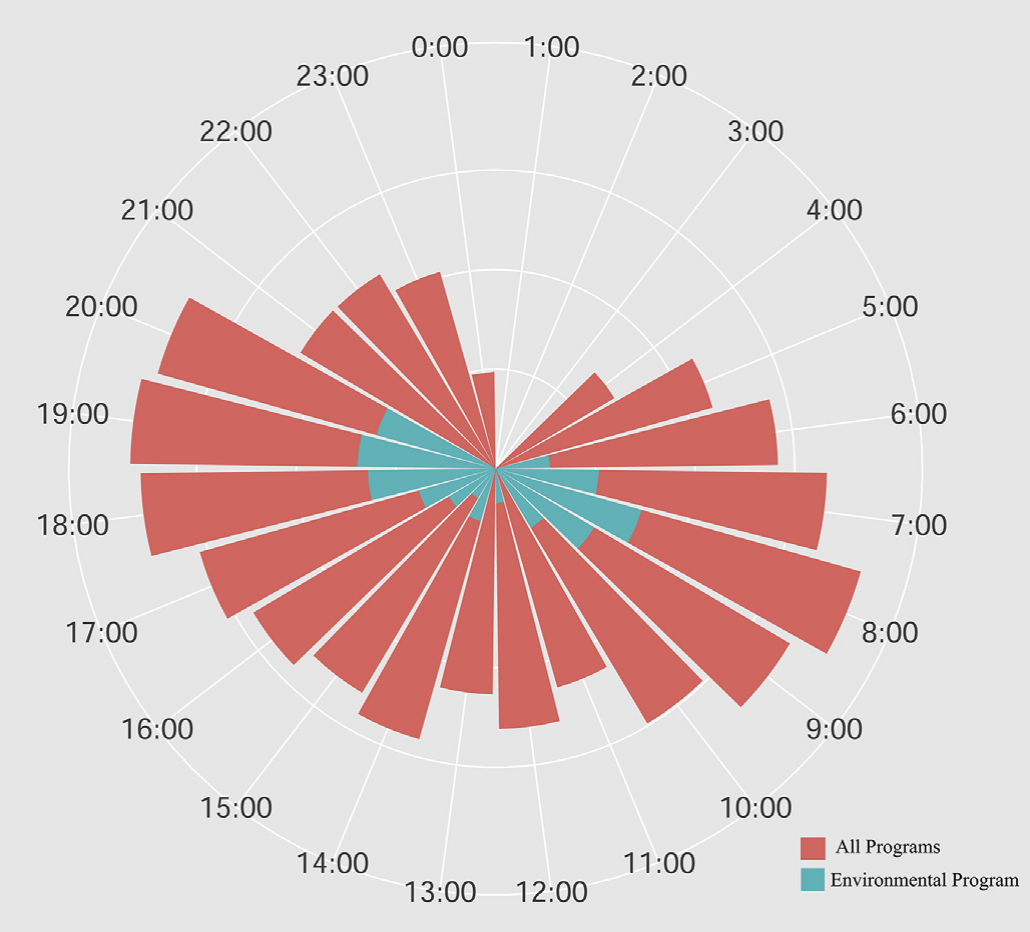
A polar plot showing frequencies of all (red) and environmental programs in a 24 hour of a day. The length of radiate bar is proportionate to the frequency of programs.

Environmental programs were mostly broadcasted weekly (73%), followed by daily (17%), monthly (8%) and fortnightly (0.64%), which spanned one to 10 years with a mean of 3.51 ± 2.72 years and a median of 3 years. Radio stations aired programs from early morning (4:00) to midnight (24:00), with a consistently high coverage (>90 % radio stations) from 5:00 to 23:00 (Figure 3). There was a significant difference on broadcasting time of all radio stations and those of environmental programs (Kruskal-Wallis χ^2^ = 22.16, df = 1, P < 0.001). Environmental programs were aired mostly during prime time (75%), (6:00–8:00) (33%) and (18:00–20:00) (42%) (Figure 3). Nearly, 75% radio stations responded that environmental issues were covered during special events (e.g., world environment day, international day for biological diversity etc.). There were, however, significant differences between radio stations that air environmental program regularly (84%) and those without regular environmental program (64%) (two proportion z-test: χ^2^ = 4.8, P = 0.02). Environment related events covered by radio stations differed significantly (χ^2^ = 74.7, df = 4, P < 0.001), with a high focus on World Environment Day (32%), Earth Day (33%), World Water Day (22%) and a low focus on Biodiversity Day (10%) and others (3%).

### 3.3 Enhancing popularity and collecting audience feedback mechanism of environmental programs

Nearly 73% radio stations conducted “Vox Pop” and/or public opinion survey (poll) on environmental issues, with higher proportion of radio stations covering regular environmental programs (81%) than those without (61%) (two proportion z-test: χ2 = 3.9, P = 0.04). Most of the radio stations (89%) reported airing jingles and public service announcement (PSA) on environmental issues. There was no significant difference in airing jingles and PSA between radio stations that had (94%) and had not (81%) regular environmental program (two proportion z-test: χ2 = 3.15, P = 0.07).

Of the total radio stations airing environmental programs, 79% used various types of audience feedback mechanisms, which differed significantly (X^2^ = 26.57, df = 4, P < 0.001). Facebook was the most reported feedback mechanism (31%), followed by short message service (30%), interactive voice response (20%), other (15%) and toll-free (4%). Nearly 75% radio stations used either one (22%), any two (23%) and any three (23%) and all (2%) of the following strategies: quiz competition, radio listeners' group, entertainment and others, to popularize environmental programs.

### 3.4 Perceived impacts of environmental program

Of radio stations that aired environmental program, 50% admitted positive impacts at local level, in which 31% were able to give some specific examples or/and cases of perceived impacts (Table 2). In some areas, school students formed Eco-clubs, in some cases people are engaged in tree plantation in their household premises while some reported reductions in fire in forests. Other changes mentioned include positive attitude of people towards protected areas, increase in open defecation free zones and improvement in hygiene behavior of general public. Further assessment is required to validate such positive impacts.

**Table 2:** Perceived impacts of environmental programs at community level (text has been edited for clarity).

Students formed Eco-clubs, and they are getting support from parents and schools.
Open defecation (*Khula Disha*) was common but it is reduced a lot.
We have noticed that people are engaged in sanitation, afforestation, and preserving the environment.
Due to our programs on environment, people are engaged in tree plantation.
It has contributed to conserve the environmental resources through awareness program. It has minimized pollution and reduced fires in jungle which were the major problems of Terai.
Our program helped to engage local people to make our society clean and green. There are few wastes in roads, and trees are growing.
People have started plantation in their house premises, as they knew the importance of trees.
People can talk about how to be safe in disaster time.
People knew importance of protected areas (*Nikunja*). They have a positive attitude towards protected areas.

### 3.5 Challenges of running environmental program

Nearly 22% of all radio stations terminated environmental program in the past, with no significant difference in proportion between community (23%) and commercial (21%) radio stations (Z-test for two proportions). We found high levels of agreement among respondents on the importance of environment program for radio stations (agreement = 0.74): all radio stations (89%), those airing environmental programs (95%) and not airing environmental programs (81%). Surprisingly, we found similar interest to broadcast environmental programs among those not airing environmental programs (9%) and those who aired environmental programs in the past (4%) (two proportion z-test: χ2 = 0.044, P = 0.8).

**Figure 4:**
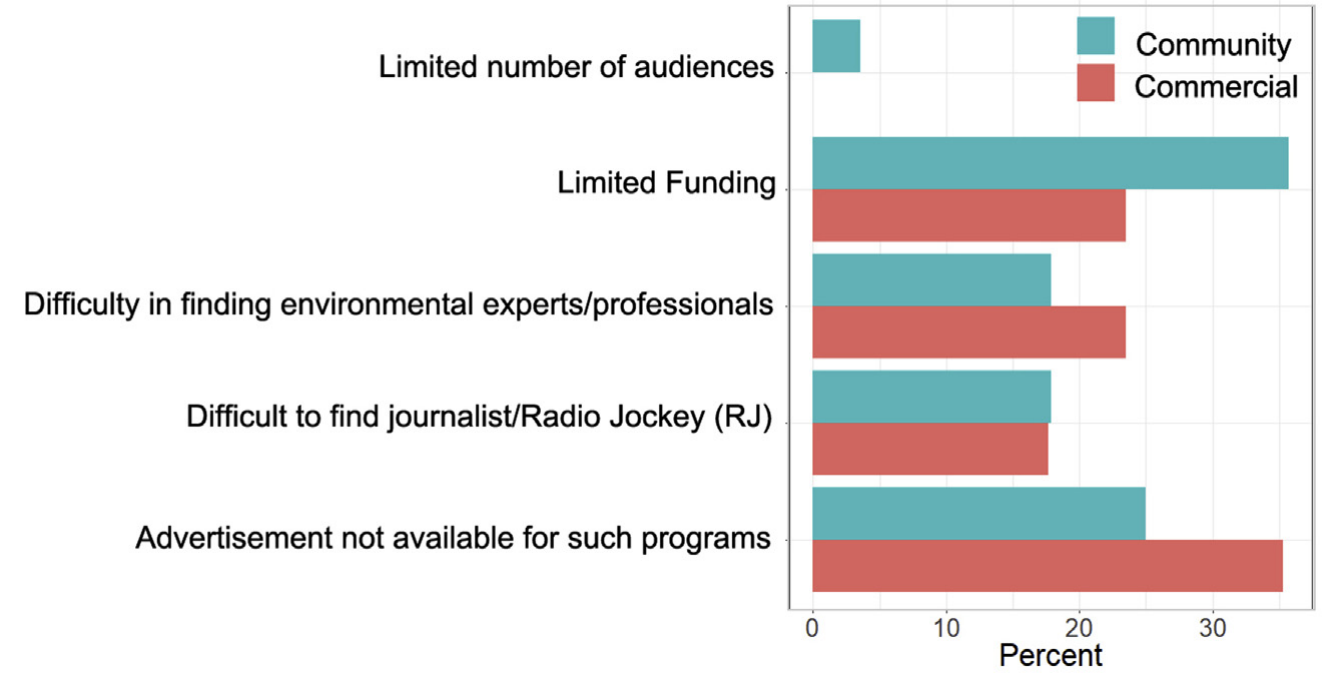
Percent of respondents that selected major causes of discontinuation of environmental programs. None of the respondents selected political interference and others.

The most common factors of discontinuity of environmental programs were lack of advertisement and funding for such programs. Limited environmental experts/professionals and radio journalists were the third most reported causes for both categories of radios. Limited number of audience and political interference were not important drivers (Figure 4).

**Figure 5:**
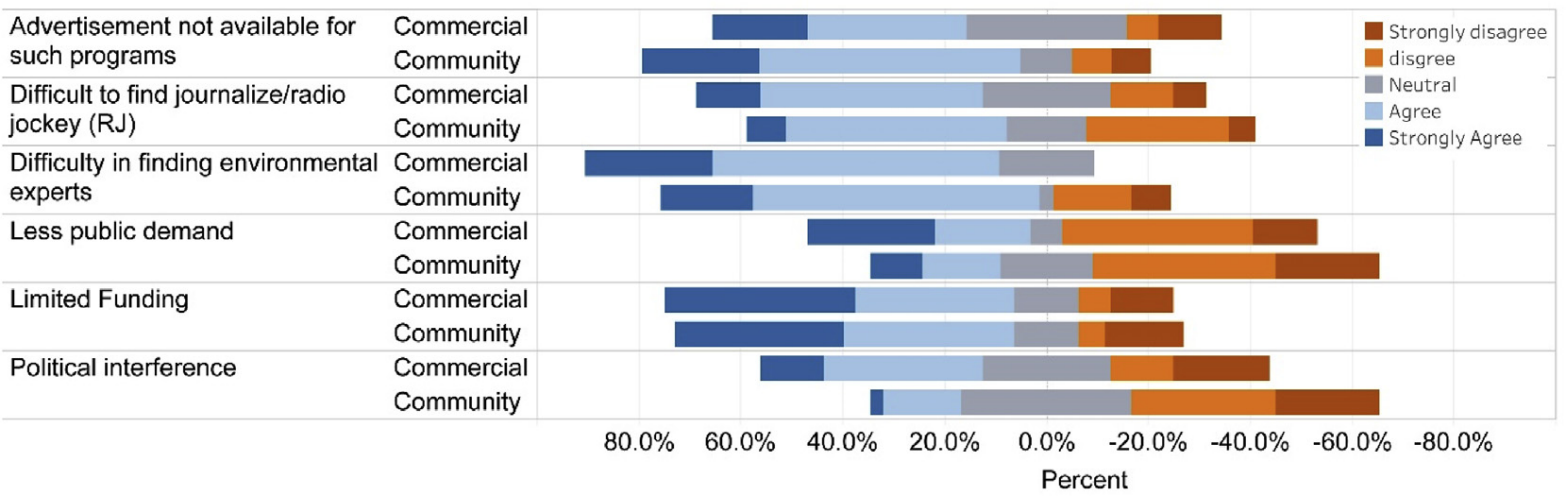
The views of respondents on key challenges in running environmental program. The distribution of responses is shown for each survey item.

Among the major challenges in running environmental programs, respondents had a diverse viewpoint. Respondents disagreed mostly on less public demand, and political interference as the major challenges, with a higher percentage of disagreement that came from community radio stations (Figure 5). Limited funding and difficulty in finding environmental experts received more agreements from respondents at both types of radio stations, with slightly more agreement from commercial radio stations.

**Figure 6:**
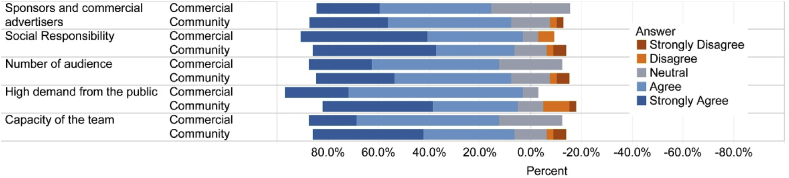
The views of respondents about the most important factors while adding new environmental programs in radio stations.

We found that most of the respondents had a high level of agreement on responses, agreeing on multiple survey items on important factors while adding new environmental programs. Respondents agreed mostly on both social responsibility (agree 82%, - 7%, neutral-11%) and high demand from public (agree -82%, disagree - 9%, 9%), followed by capacity of team (agree - 78%, disagree - 16%, neutral -5%), sponsors and commercial advertisers (agree - 76%, disagree - 4% and neutral - 20%), number of audience (agree 76%, disagree - 18%, neutral - 5%). However, there are variations between community and commercial radio stations. Social responsibility, number of audiences, high demand from public and capacity of the team were important for commercial radio stations, whereas sponsors and commercial advertisers were important for community radio stations (Figure 6).

### 3.6 Funding sources of environmental program

**Figure 7:**
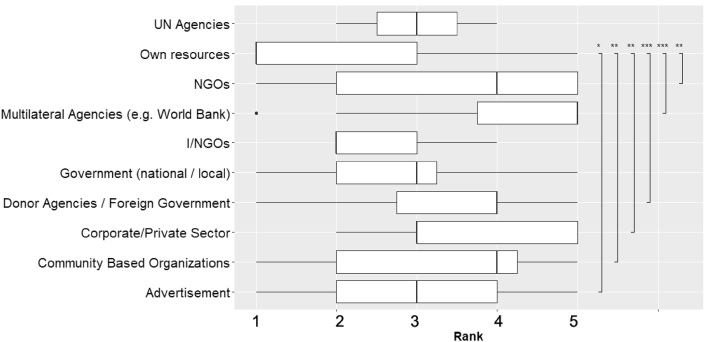
Box plot of ranks of various funding sources to run environmental programs. The higher rank suggests the higher importance. The boundary of the box indicates the 25^th^ (lower) and 75^th^ (upper) percentile. A solid line within box marks median. The whiskers above and below the box indicate the variability outside the upper and lower quartiles. Plots with ∗ indicates a significant difference in post-hoc multiple comparison tests (∗— ≤ 0.05, ∗∗ — ≤ 0.01, ∗∗∗— ≤ 0.001).

There is a significant difference between the ranks of different funding sources (Kruskal-Wallis χ^2^ = 46.15, df = 9, P < 0.0001). Dunn's multiple comparisons tests suggested own resources were significantly different from NGOs, multilateral agencies, donor agencies/foreign governments, corporate/private sector, community-based organizations and advertisement (Figure 7). The main funding sources were own resources and I/NGOs (Figure 7). However, there were considerable variations in the reported funding sources when not accounting for rank. Funding from government (national/local) (18%) and own sources (18%) were reported most often, followed by advertisement (17%) and donor agencies/foreign government (12).

## 4 Discussion

### 4.1 Radio channels and coverage of environmental programs

This study provides the first nation-wide assessment of coverage of environmental issues in radio stations, including challenges while running environmental programs. Our survey showed that radio stations in Nepal had large coverage in terms of districts (both own network and associated networks) and audience size (Table 1), suggesting their popularity [45], similar to Africa [46], Asia and the Pacific [20]. In Nepal, the number of radio stations increased from 50 in 2005 [47] to 740 in 2017, of which, most of them were established around 2011. Since almost all radios had internet live stream and were associated with a network, radio virtually had a global coverage via mobile app and internet. With 40 million mobile phone subscribers, a rapid expansion of internet coverage and improvements in bandwidth speed [48], radio listeners might have surged in Nepal among those who do not prefer using traditional ‘radio’.

We found that environmental programs were covered in 58% radio stations who participated in our survey, but environment related programs were among the least popular radio programs. Our study suggests entertainment and general were among the most popular categories of radio programs. This is plausible as entertainment has power to meet human psychological needs and touches people [49]. People have produced and enjoyed music throughout human history and across all cultures [50]. Thus, pleasure-seeking human behavior affects in determining media content, and informational utility becomes the secondary choices [51, 52].

Of the total radios who participated in this survey, 65% were community radios. Community radios basically are managed and owned by a community, and their operations reflect their special interests and needs of the community [22]. Our results showed a high priority of environmental issues in community radio stations. It is, however, far less than other categories of programs that community radio airs. Community radios have grown in numbers worldwide offering education for and access to communicate issues of grassroots populations, including local context-specific environmental issues [22, 53]. Studies have shown that many community radios face sustainability crisis as funding dries up, and their intended purposes consequently get less prioritized to survive [54, 55]. This may be true in Nepal as our results suggest that that 23% of community radio channels discontinued environment related programs and none of them were interested in rebroadcasting it. We acknowledge that there are no specific criteria to determine the sufficiency of media coverage of environmental issues in the community radio stations.

### 4.2 Looking inside into environmental program

Although environmental programs covering general category were the most common in both types of radio stations, the commercial radio stations aired a higher proportion of such programs (~70%) than community radios (~50%). The community radios focused more often on specialized environmental programs (e.g., climate change, disaster risk management, and forest and wildlife conservation) (Figure 2). Interestingly, all these specialized programs were reported as being less popular than their shares except for water resource management (Figure 2). There are several reasons for the low coverage of specific environmental programs in the radio stations. First, the production of media content on a specific environmental issue is both expensive and time-consuming as it requires (a) journalists specialized in environmental reporting, (b) funding for investigation and (c) support and availability of subject experts [56, 57, 58]. Second, environmental issues presented in good stories with local connections are well received by community [38]. The high coverage and popularity of radio program on forest and wildlife conservation and water resource management may be the result of local connection. The majority of Nepalese people live in rural areas where the forest is an integral part of subsistence livelihoods [59]. The same holds for water resources, which not only sustain rain-fed agriculture, but they are eventually linked to health and sanitation [60].

Environmental programs were aired mostly during prime time on a weekly basis. Business enterprises target prime time to reach a maximum number of audiences and usually pay a high price to run their advertisements. This is an indication of priority given to the environmental programs. Studies have shown that airing prime time commercials has significant positive changes in raising public awareness in several cases [61]. Interestingly, coverage of environmental issues during special events was high in both radio stations covering regular environmental issues (81%) and without (61%). Such prominence given to environmental events is a product of the influence of environmental activism fueled by globalization, publicity and campaigns by global environmental organizations [62]. Such a relatively infrequent coverage has limited impact, despite some remarkable exceptions [63]. The high coverage of World Environment Day might be a result of active participation of government agencies, non-government organizations and academia, to celebrate these global outreach campaigns on a broad subject, appeal to emerging environmental issues such as pollution, climate change, wildlife conservation and sustainable livelihoods. Other environment related events are very specific and thus may have received less attention. During such events, most of the radio stations used various means of collecting public opinion such as vox pop or/and public opinion survey to connect directly with audience and make connection. Such a short term—once a year—media attention has been reported being very ineffective [64].

### 4.3 Impacts of media coverage at local level

Nearly 50% of radio stations that aired environmental program reported positive impacts of environmental coverage at local level. Some of the reported notable impacts were the formation of eco-clubs, tree plantation and reduction of open defecation. Studies have shown that media have been effective in bringing people's behavior change and making an impact at local levels [3, 15, 35, 65]. However, the effectiveness of mass media in changing human attitudes and behavior depends on how targeted and well-executed media campaigns are [66]. Positive impacts attributed to radios in our results, however, may not solely be attributed only to radio programs although radios are very influential in expanding public awareness on environmental issues [36]. Further assessment is needed to ascertain the extent of impact brought by mass media in Nepal.

### 4.4 Challenges of running environmental program

Our results suggested that the cease of environmental programs in both community and commercial radio stations were mostly due to limited funding (advertisement, program sponsor) (Figure 4). Other problems included difficulty in finding environmental experts/professionals and radio journalists although they were reported less frequently (Figure 4). Among those radio stations airing environmental programs, also expressed similar challenges in running environmental programs although there are some variations between community and commercial radio stations. Funding is a common challenge for public media worldwide [67], similar to our findings. Community radio stations often face restrictions such as the use of local language only and barring from advertisement selling and broadcasting news [46]. Nepal's community radios do not face such restrictions. It is, however, important to note that both community and commercial radio stations had similar responses on the prerequisite of adding environmental programs. Community radios are expected to show a significantly high interest in running environmental programs. It is therefore likely that community radios may have deviated from their core values although we acknowledge that they have restrictive resources. The Association of Community Radio Broadcasters in Nepal (ACORAB), a national umbrella organization of community radios, requires community radios be run by non-profit making organization, broadcasting program on education, health and development for at least 40% of its airtime; and broadcasting 60% of total programs locally [27]. Several questions surfaced regarding the compliance of these requirements, and community radios are blamed for being suffered from elite capture, loss of local connection and accountability [27]. Elite capture happens when an individual with disproportionate access to social, political or economic power uses his/her leverage for a position in a community-based organization and its decision.

Our results suggested that own resource is the top-ranked funding source, followed by INGOs. UN agencies, government (national/local), corporate/private sector and advisements constituted the third most important funding sources. It implies that environmental programs are mostly self-funded and face financial uncertainty. Nepal is one of the highest recipients of foreign aid in South Asia, where a large proportion of such aid goes into social services (e.g., education, hospitals, water supply), followed by transport, power and communications sectors, including agriculture sector [68]. Radio stations were beneficiaries of foreign aid and support from international organizations. It is, however, irregular, which may put radios, particularly community radios, at high risk.

Apart from funding, lack of trained journalists and difficulty in finding environmental experts were the major challenges in running environmental programs. Local radio stations must work with limited funding and therefore rely on multi-tasking journalists and/or sharing programs through network which may lack local tastes and values [69]. In Nepal, most of the radio stations were associated with network, programs sharing, including those of environmental ones, may be common. In developed countries, journalists with science credentials and/or trained journalists specialized for science reporting are highly sought in big mass media [70]. Such situations may not be practically feasible in Nepal not only due to limited funding but also due to limited experts in the field.

## 5 Conclusion and recommendations

Radios have been the most preferred means of communication worldwide and media campaigns on social issues are popular [65, 71]. In Nepal, radio stations are popular, accessible across the country and cover a wide array of public concerns including environmental issues [45]. We conclude that environmental programs were included in the majority of Nepal's FM radios and integrated into prime airtime, but environmental programs were neither preferred ones for radio operators nor the general public. Such unexpected results may have emanated from lack of trained journalists and subject experts, limited funding and lack of institutional priority. Since Nepal is a predominately agrarian and mountainous country where both poverty (poverty headcount rate – 25.2%) and illiteracy (adult illiteracy rate – 40.43%) are acute [72], we argue that there is no viable alternative to FM radios for environmental communication in the country. We recommend the Government of Nepal to formulate a media engagement strategy to make environmental programs self-sustainable, inclusive, representative and popular. The strategy will be pivotal for public awareness in Nepal, including a mechanism for providing support, as identified by our study, for training journalists, providing funds and connecting with scientists/experts. It requires commitments and concerted efforts from governments (e.g., federal, state and local), both community and commercial radio networks, academia, and donor communities.

## Declarations

### Author contribution statement

P.K. Paudel: Conceived and designed the experiments; Performed the experiments; Analyzed and interpreted the data; Contributed reagents, materials, analysis tools or data; Wrote the paper.

R. Bastola: Conceived and designed the experiments; Performed the experiments; Contributed reagents, materials, analysis tools or data; Wrote the paper.

P.T. Lopchan: Contributed reagents, materials, analysis tools or data.

### Funding statement

This work was supported by the Kathmandu Institute of Applied Sciences, Kathmandu, Nepal (Grant Number: 2018-02).

### Competing interest statement

The authors declare no conflict of interest.

### Additional information

No additional information is available for this paper.
