# Supplementary material for: The coverage of environmental issues in FM radios in Nepal: the current status and challenges
Source: Heliyon. 2020 Jul 3;6(7):e04354. doi: 10.1016/j.heliyon.2020.e04354 (PMC7339057; doi:10.1016/j.heliyon.2020.e04354)
Supplement: Appendix 1 [file mmc1.pdf]

Supporting Information for

The coverage of environmental issues in FM radios in Nepal: The current status and challenges

Paudel et al. (2020). Heliyon

Appendix 1: Sample of questionnaire survey on "The coverage of environmental issues in FM radios in Nepal: The current status and challenges"

Kathmandu Institute of Applied Sciences

* Required Information

Status of Environmental Issues in Mass Media

page 1

* 1.

Dear Station Manager:

Greetings from Kathmandu Institute of Applied Sciences (KIAS), Kathmandu, Nepal!

As you know, mass-media particularly FM radio, has an important role in defining and shaping environmental problems in the modern society. In this context, we are conducting this survey to understand mass-media coverage on environmental issues in Nepal and its influence on public awareness. Your time and expertise is far more valuable to understand these issues. The survey is very brief and will only take about 15 minutes to complete.

Your responses will be coded; therefore, information entered will remain anonymous. Data from this research will be reported only in aggregate in the form of scientific reports (e.g., journal articles, research communication, and policy notes etc.). The KIAS Institutional Review Board has approved this survey.

If you have any questions, please contact us:

Dr. Prakash K. Paudel (pkpaudel@kias.org.np), Scientist, Kathmandu Institute of Applied Sciences, Kathmandu, Nepal

Mr. Rabin Bastola, (rabinbastola@gmail.com), Assistant Professor, Department of Environmental Science, Amrit Campus, Tribhuvan University, Kathmandu, Nepal

Mr. PT Lopchan, (journalistpt@gmail.com), Senior Reporter, Ujyaalo Radio Network, Lalitpur, Nepal

Thank you very much for your time and cooperation.

Please kindly start the survey by clicking on the "Agree" and then "Next" button.

(Select one option)

- ○ Agree

Kathmandu Institute of Applied Sciences (www.kias.org.np)

Status of Environmental Issues in Mass Media

page 2

*** 2. Please provide information of your radio station?**

Name (Radio Call Sign)

____

____

*** 3. Type (Select one option)**

- ○ Commercial
- ○ Community

*** 4. Address**
(e.g, Sundarbazar Municipality-3, Lamjung)

____

____

*** 5. Radio Frequency (MHz)**

____

____

*** 6. Transmitter Power (Watt):**

____

____

7. Coverage (No. of Districts):

	0	1	2	3	4	5	6	7	8	9	10	11	12	13	14	15	16	17	18	19	20	21	22	[illegible]
*(a) Own station coverage	○	○	○	○	○	○	○	○	○	○	○	○	○	○	○	○	○	○	○	○	○	○	○	○
*(b) Network coverage	○	○	○	○	○	○	○	○	○	○	○	○	○	○	○	○	○	○	○	○	○	○	○	○

*** 8. Estimated Number of Audience:**

____

____

*** 9. Established Year**

____
____

*** 10. Broadcast duration in a day (in hours). (Select one option)**

- ◯ 1
- ◯ 2
- ◯ 3
- ◯ 4
- ◯ 5
- ◯ 6
- ◯ 7
- ◯ 8
- ◯ 9
- ◯ 10
- ◯ 11
- ◯ 12
- ◯ 13
- ◯ 14
- ◯ 15
- ◯ 16
- ◯ 17
- ◯ 18
- ◯ 19
- ◯ 20
- ◯ 21
- ◯ 22
- ◯ 23
- ◯ 24

*** 11. Broadcast time (e.g. 6 AM-10 AM)** [Answer this question only if answer to Q#10 is 1 OR 2 OR 3 OR 4 OR 5 OR 6 OR 7 OR 8 OR 9 OR 10 OR 11 OR 12 OR 13 OR 14 OR 15 OR 16 OR 17 OR 18 OR 19 OR 20 OR 21 OR 22 OR 23]

* 12. Available on internet (live streaming / downloadable version) (Select one option)

- ◯ Yes
- ◯ No

*** 13. Associated Radio Network**
Please check all applicable fields

- ☐ Community Information Network (CIN) of the ACORAB
- ☐ Kantipur FM
- ☐ Nepal FM
- ☐ Ujyaalo 90 Network (UNN)
- ☐ Other (Please specify) ________________

Status of Environmental Issues in Mass Media

14. Please list five most popular programs (EXCEPT NEWS) aired by your radio station?

* (a) First Most Popular

(b) Second Most Popular

(c) Third Most Popular

(d) Fourth Most Popular

____
____

(e) Fifth Most Popular

____
____

15. Select appropriate theme of program

	Economy	Entertainment	Environment	Health	Politics	Science and Technology	Society	Sports	Above all
*(a) First Most Popular	○	○	○	○	○	○	○	○	○
(b) Second Most Popular	○	○	○	○	○	○	○	○	○
(c) Third Most Popular	○	○	○	○	○	○	○	○	○
(d) Fourth Most Popular	○	○	○	○	○	○	○	○	○
(e) Fifth Most Popular	○	○	○	○	○	○	○	○	○

*** 16. Do you broadcast program(s) on environmental issues these days? (Select one option)**

○ Yes Go to Page No. 8

○ No Go to Page No. 5

Status of Environmental Issues in Mass Media

*** 17. Did you broadcast program(s) on environmental issues in past? (Select one option)**

- ○ Yes Go to Page No. 6
- ○ No Go to Page No. 21

Status of Environmental Issues in Mass Media

*** 18. Why did you discontinue program(s) on environmental issues? (please check all applicable)**

- ☐ Limited funding
- ☐ Advertisement not available for such programs
- ☐ Difficult to find journalist/Radio Jockey (RJ)
- ☐ Difficulty in finding environmental experts/professionals (while running many episodes)
- ☐ Limited number of audiences
- ☐ Political interference
- ☐ Other (Please specify) ________________

Go to Page No. 21

Status of Environmental Issues in Mass Media

*** 19. Do you have reporter/correspondent on environmental issues? (Select one option)**

○ Yes Go to Page No. 9

○ No Go to Page No. 9

*** 20. How many reporters/correspondents are working in your FM station on environmental issues? (Select one option)**

○ 1
○ 2
○ 3
○ 4
○ 5
○ 6
○ 7
○ 8
○ 9
○ 10
○ >10

Kathmandu Institute of Applied Sciences (www.kias.org.np)

Status of Environmental Issues in Mass Media

Please provide details of your radio program(s) related to environmental issues.

21. Name of Program

* (a) 1

____
____

(b) 2

____
____

(c) 3

____
____

(d) 4

____
____

(e) 5

____
____

22. Focus Area

	Environmental Pollution	Forest and Wildlife Conservation	Climate Change	Disaster Risk Management	Water Resource Management	General
*(a) 1	○	○	○	○	○	○
(b) 2	○	○	○	○	○	○
(c) 3	○	○	○	○	○	○
(d) 4	○	○	○	○	○	○
(e) 5	○	○	○	○	○	○

23. Time (e.g. 8.30AM)

* (a) 1

____
____

(b) 2

____
____

(c) 3

____
____

(d) 4

____
____

(e) 5

____
____

24. Frequency

	Daily	Weekly	Fortnightly	Monthly
*(a) 1	○	○	○	○
(b) 2	○	○	○	○
(c) 3	○	○	○	○
(d) 4	○	○	○	○
(e) 5	○	○	○	○

25. How long? (No. of year)

	<1	1	2	3	4	5	6	7	8	9		
*(a) 1	○	○	○	○	○	○	○	○	○	○	○	○
(b) 2	○	○	○	○	○	○	○	○	○	○	○	○
(c) 3	○	○	○	○	○	○	○	○	○	○	○	○
(d) 4	○	○	○	○	○	○	○	○	○	○	○	○
(e) 5	○	○	○	○	○	○	○	○	○	○	○	○

Which is the most effective program on environmental issue?

26. Name of Program

***** (a) First Most Popular

____
____

(b) Second Most Popular

____
____

(c) Third Most Popular

____
____

27. Reason

(a) First Most Popular

____
____

(b) Second Most Popular

____
____

(c) Third Most Popular

____
____

28. What are the most important factors while adding new environmental programs in your radio?

	Strongly Agree	Agree	Neutral	Disagree	Strongly Disagree
*(a) Sponsors and commercial advertisers	○	○	○	○	○
*(b) Number of audience	○	○	○	○	○
*(c) Capacity of the team	○	○	○	○	○
*(d) High demand from the public	○	○	○	○	○
*(e) Social Responsibility	○	○	○	○	○

Status of Environmental Issues in Mass Media

*** 29. Are local issues included in the environmental program? (Select one option)**

- ◯ Yes
- ◯ No

Status of Environmental Issues in Mass Media

*** 30. Which of the following are the main funding sources of your programs on environmental issues?**
Please rank exactly 5 options. **[Please rank exactly 5 option(s).]**

Own resources	
Advertisement	
Government (national / local)	
Donor Agencies / Foreign Government	
Multilateral Agencies (e.g. World Bank)	
UN Agencies	
NGOs	
I/NGOs	
Corporate / Private Sector	
Community Based Organizations (CBOs)	

Status of Environmental Issues in Mass Media

page 14

*** 31. Do you use audience feedback mechanisms for environmental programs? (Select one option)**

- ○ Yes — Go to Page No. 15
- ○ No — Go to Page No. 16

Status of Environmental Issues in Mass Media

page 15

*** 32. What kind of audience feedback mechanisms are in place for environmental programs?**

- ☐ Interactive Voice Response (IVR)
- ☐ SMS
- ☐ Facebook page
- ☐ Toll-Free Number
- ☐ Other (Please specify) ________________

Kathmandu Institute of Applied Sciences (www.kias.org.np)

Status of Environmental Issues in Mass Media

page 16

*** 33. Do you take any approaches to popularize environmental programs? (Select one option)**

- ○ Yes — Go to Page No. 17

○ No Go to Page No. 18

Status of Environmental Issues in Mass Media

page 17

*** 34. Which approach do you take to popularize environmental programs?**

- [ ] Quiz competition (with gift hamper)
- [ ] Formation of Radio Listeners' Club
- [ ] Including entertainment component
- [ ] Other (Please specify) _______________

Status of Environmental Issues in Mass Media

page 18

*** 35. Have you documented any positive impacts (at local level) of environmental program aired by your radio? (Select one option)**

○ Yes Go to Page No. 19

○ No Go to Page No. 20

Kathmandu Institute of Applied Sciences (www.kias.org.np)

Status of Environmental Issues in Mass Media

page 19

36. Please provide details of the impact

____

____

37. What are the major challenges to produce and/or broadcast programs on environmental issues?

	Strongly Disagree	Disagree	Neutral	Agree	Strongly Agree
*(a) Limited funding	○	○	○	○	○
*(b) Advertisement not available for such programs	○	○	○	○	○
*(c) Difficult to find journalist/Radio Jockey (RJ)	○	○	○	○	○
*(d) Difficulty in finding environmental experts	○	○	○	○	○
*(e) Less public demand	○	○	○	○	○
*(f) Political interference	○	○	○	○	○

*** 38. Do you produce and/or broadcast programs on environmental issues during special events/days like the World Environment Day etc.? (Select one option)**

○ Yes Go to Page No. 22

○ No Go to Page No. 23

Status of Environmental Issues in Mass Media

*** 39. Which special events/days are often considered for? (check all applicable)**

- ☐ World Water Day (22 March)
- ☐ Earth Day (22 April)
- ☐ International Day for Biological Diversity (22 May)
- ☐ World Environment Day (5 June)
- ☐ Other (Please specify) _______________

Status of Environmental Issues in Mass Media

*** 40. Do you conduct "Vox Pop" and/or public opinion survey (poll) on environmental issues? (Select one option)**

- ○ Yes
- ○ No

Kathmandu Institute of Applied Sciences (www.kias.org.np)

Status of Environmental Issues in Mass Media

*** 41. Do you air radio jingles and Public Service Announcement (PSA) on environmental issues? (Select one option)**

- ○ Yes Go to Page No. 25
- ○ No Go to Page No. 27

42. In which of following thematic areas do you produce and/or broadcast radio jingles and Public Service Announcement (PSA) ?

	produce and broadcast	only broadcast	neither produce nor broadcast
*(a) Environmental Pollution	○	○	○
*(b) Forest and Wildlife Conservation	○	○	○
*(c) Climate Change	○	○	○
*(d) Disaster Risk Management	○	○	○
*(e) Water Resource Management	○	○	○

*** 43. In what frequency do you broadcast such jingle / PSA?**

- ☐ Daily
- ☐ During special occasion/day (e.g. World Water Day)
- ☐ During particular season
- ☐ Depends on external support (funding)
- ☐ Other (Please specify) _______________

Status of Environmental Issues in Mass Media

page 27

*** 44. How important is environmental program for your radio?** (Select one option)

○ Very Important ○ Important ○ Neutral ○ Fairly Important ○ Not important

Status of Environmental Issues in Mass Media

page 28

*** 45. Is there any demand for programs on environmental issues from general public? (Select one option)**

○	Yes	Go to Page No. 29
○	No	Go to Page No. 30

Kathmandu Institute of Applied Sciences (www.kias.org.np)

Status of Environmental Issues in Mass Media

page 29

46. Please rank each theme by the level of public demand.

	Very Important	Important	Moderately Important	Slightly Important	Not Important
*(a) Environmental Pollution	○	○	○	○	○
*(b) Forest and Wildlife Conservation	○	○	○	○	○
*(c) Climate Change	○	○	○	○	○
*(d) Water Resource Management	○	○	○	○	○
*(e) Disaster Risk Management	○	○	○	○	○

Status of Environmental Issues in Mass Media

*** 47. Do you perform monitoring and evaluation of your program(s)? (Select one option)**

- ◯ Yes — Go to Page No. 31
- ◯ No — Go to Page No. 32

Status of Environmental Issues in Mass Media

*** 48. When do you perform evaluation of your program(s)? (Select one option)**

- ◯ Yearly — Go to Page No. 33
- ◯ Every Five Year — Go to Page No. 33
- ◯ While adding new programs — Go to Page No. 33
- ◯ Depends on funding — Go to Page No. 33
- ◯ Other (Please specify) ___________ Go to Page No. 33

Kathmandu Institute of Applied Sciences (www.kias.org.np)

Status of Environmental Issues in Mass Media

*** 49. Do you have plans to broadcast program related to environmental issues? (Select one option)**

- ◯ Yes
- ◯ No

Status of Environmental Issues in Mass Media

*** 50. Are you interested to relay/re-broadcast environmental program produced by other agencies? (Select one option)**

- ◯ Yes
- ◯ No

Status of Environmental Issues in Mass Media

*** 51. Would you like us to contact you in case we might have more questions for this research?** (Select one option)

- ◯ Yes Go to Page No. 35
- ◯ No Go to Page No. 36

Status of Environmental Issues in Mass Media

*** 52.**
Please write your email address below.

____
____

53. Please provide us your personal information

*(a)	Gender	:	○ Male<br>○ Female
*(b)	Age	:	
*(c)	Academic qualification	:	
*(d)	Position	:	
*(e)	Work experience in radio	:	
