# Supplementary material for: The coverage of environmental issues in FM radios in Nepal: the current status and challenges
Source: Heliyon. 2020 Jul 3;6(7):e04354. doi: 10.1016/j.heliyon.2020.e04354 (PMC7339057; doi:10.1016/j.heliyon.2020.e04354)
Supplement: Supplementary file 2 — Appendix 2 [file mmc2.docx]

**Supporting Information for**

*The coverage of environmental issues in FM radios in Nepal: The current status and challenges*

*Paudel et al. (2020). Heliyon*

| **Appendix 2: List of names of environmental programs by category** | |
| --- | --- |
| **Program Name** | **Category** |
| Hamro Batabaran | General |
| Eco Sansar | General |
| Jagat Bigyan | General |
| Good Morning Bindas | General |
| Jalapa Khabar | General |
| Hamro Batawaran | General |
| Parkirti ra Ma | General |
| Reed + | Climate Change |
| Abhiyan Quiz | General |
| Swastha ra Sarsafai | General |
| Pani ra Sarsafai | Water Resource Management |
| Ban tatha Batabaran | Forest and Wildlife Conservation |
| KHABAR RAFTAR | General |
| Bhukampako Byathaa | General |
| Khetiwari | Water Resource Management |
| NAGAR GATIBIDHI | General |
| Madhyawarti Awaj | Forest and Wildlife Conservation |
| Jalbayo Paribartan | Climate Change |
| Jana Sawal | General |
| Hamra kura | General |
| Paryawaran | General |
| Arun Ko Sarofafo | General |
| Paryabaran | General |
| Krishi Chautari | Water Resource Management |
| jibanka lagi ban | Forest and Wildlife Conservation |
| Forest Conservation and Wildlife Reserve | Forest and Wildlife Conservation |
| Hamro Ban | Forest and Wildlife Conservation |
| Khanepani | Water Resource Management |
| Environmental,Health and Education | General |
| Hamro Batabaran | General |
| Swasthya Sikshya | General |
| Environmental Polution | Environmental Pollution |
| Water Resource Management | Water Resource Management |
| Hamro Swasthya | General |
| Harit Nagar | General |
| **Program Name** | **Category** |
| BKP | General |
| Batabaran | General |
| Koseli Bahasa | General |
| ban tatha batabaran | Forest and Wildlife Conservation |
| Swasthya ra Sansar | General |
| Swasthya Sandesh | General |
| Ban Batabaran | Forest and Wildlife Conservation |
| Jibika | General |
| Hamro Bikash | General |
| About Climate | Climate Change |
| Wish you all the best | General |
| Simsar | Forest and Wildlife Conservation |
| ODF programme | Water Resource Management |
| Paurakhi Bahas | General |
| Bhannichhin Aama | General |
| Janasambadh | General |
| Jajarkot Bisesh | General |
| sAMaya sandarva | General |
| Ban batika | Forest and Wildlife Conservation |
| Sarsafai Bahas | Water Resource Management |
| Samrakshad ma Sagasagai | General |
| Focus | General |
| Lok Dohori | General |
| Jalabayu Paribartan | Climate Change |
| Climate Change and Agriculture | Climate Change |
| Sarokar | General |
| Swastha Sansar | General |
| Sarsafai | General |
| Ganthan Manthan | General |
| Waste Management | Environmental Pollution |
| Paila Bisesh | General |
| Jalabayou Sachetana | Climate Change |
| Sociaty | General |
| t.t.s. Gatibidhi | General |
| Water resource Management | Water Resource Management |
| Bishesh Bahas | General |
| Paryatak ko Bato | General |
| ban ra banaspati | Forest and Wildlife Conservation |
| Hamro TilottAMa | General |
| Youth | General |
| **Program Name** | **Category** |
| Ban Ra Jiban | Forest and Wildlife Conservation |
| Indreni quiz | General |
| Gidda Samrakshyan | Forest and Wildlife Conservation |
| Climate Change | Climate Change |
| Upparamukh sanga Nagargatibid | General |
| Aaj ko Sandarbha | General |
| prakop | Disaster Risk Management |
| Ban Kariya Natak | General |
| Janta ke Aawaj | General |
| Srijana ka Fool Haru | General |
| JhulkeghAM | General |
| Dristikon | General |
| Sadak Nirman | Environmental Pollution |
| Hamro Jalbayu | Climate Change |
| Bhukampiya Surakshya | Disaster Risk Management |
| Youth | General |
| Forest and Wildlife conservation | Forest and Wildlife Conservation |
| Quiz Time | General |
| Hamro Batabaran | General |
| Jana Boli | General |
| Hamro Swasthe | General |
| Indreni sarokar | General |
| Van jiwan | Forest and Wildlife Conservation |
| Bhukampiya Suraksha | Disaster Risk Management |
| Baliyo Gahar | Disaster Risk Management |
| Jal Jeewan | Water Resource Management |
